# Catastrophizing as a Predictor for Pain Perception and Disability Among Patients Undergoing Spinal Cord Stimulation

**DOI:** 10.3390/medicina61010141

**Published:** 2025-01-16

**Authors:** Juan Vicente-Mampel, Felipe Hernández-Zaballos, Francisco Javier Falaguera-Vera, David Sánchez-Poveda, Eloy Jaenada-Carrilero, Borja Huertas-Ramírez, Francisco Jose Sánchez-Montero

**Affiliations:** 1 Medicine and Health Science School, Department of Physiotherapy, Catholic University of Valencia, 46001 Torrent, Spain; juan.vicente@ucv.es (J.V.-M.); fj.falaguera@ucv.es (F.J.F.-V.); borja.huertas@mail.ucv.es (B.H.-R.); 2 Anaesthesiology Service, Pain Unit, Complejo Asistencial Universitario de Salamanca (CAUSA), 37007 Salamanca, Spain; felipezaballos@gmail.com (F.H.-Z.); sanchezpovedadavid@gmail.com (D.S.-P.); francisjsm@hotmail.com (F.J.S.-M.)

**Keywords:** chronic pain, spinal cord stimulation, catastrophization, somatosensory disorder

## Abstract

*Background and Objectives*: The International Society for Modulation defines persistent spinal pain syndrome type 2 (PSPS-type 2), formerly known as failed back surgery syndrome, as a condition where patients continue to experience pain or develop new pain following spinal surgery intended to alleviate back or lower-limb discomfort. PSPS-type 2 is characterized by pain and significant disability, affecting quality of life. Spinal cord stimulation has proven effective in treating this syndrome, although the role of psychological factors, such as pain catastrophizing and central sensitization, remain unclear. This study seeks to examine the potential connection between psychosocial responses and both functionality and pain perception in patients with persistent spinal pain syndrome type 2 who have undergone spinal cord stimulation treatment. *Materials and Methods*: A single-site, cross-sectional study was conducted on individuals diagnosed with persistent spinal pain syndrome type 2 who were receiving spinal cord stimulation. Study participants were required to meet specific eligibility criteria and were assessed for disability, pain perception, fear of movement, pain catastrophizing, and central sensitization. The spinal cord stimulation procedure involved the placement of electrodes at vertebral levels T8–T11 for precise pain control, with a particular focus on targeting the dorsal root ganglion to alleviate chronic pain. *Results*: Thirty-seven patients with persistent spinal pain syndrome type 2 have undergone spinal cord stimulation treatment for 4.68 ± 5.25 years. Clinical assessments indicated a pain perception score of 5.6 ± 1.96, Central Sensitization Inventory score of 42.08 ± 18.39, disability score of 37.62 ± 16.13, fear of movement score of 33.11 ± 8.76, and pain catastrophizing score of 28.43 ± 13.14. Finally, pain catastrophizing was significantly associated with pain perception (β = 0.075 and *p* = 0.008) and disability (β = 0.90 and *p* < 0.01). *Conclusions*: Catastrophizing plays a crucial role in pain perception and disability among patients with persistent spinal pain syndrome type 2 receiving spinal cord stimulation. Integrating psychological interventions may improve clinical outcomes for these patients.

## 1. Introduction

The diagnostic label “failed back surgery syndrome” has been deemed inaccurate and potentially problematic. In clinical practice, persistent spinal pain syndrome (PSPS) is considered the current nomenclature, with subtypes PSPS-type 1 and PSPS-type 2. PSPS is characterized by chronic low back pain, severe disability, diminished quality of life, and elevated rates of unemployment [1]. It is classified into two subtypes: PSPS-type 1, which occurs without prior spinal surgery, and PSPS-type 2, which manifests as chronic persistent pain following spinal surgery [2,3,4]. Specifically, PSPS-type 2 encompasses a diverse array of low back pain with indeterminate etiology. Consequently, PSPS-type 2 represents a syndrome with multiple causative factors and is distinguished by a significant heterogeneity among affected individuals [4,5]. A key criterion in diagnosing PSPS-type 2 is the patient’s ongoing complaint or dissatisfaction post-surgery, particularly regarding the persistence of these symptoms [4,5,6,7]. However, accurately estimating the prevalence of PSPS-type 2 is challenging, as many studies have focused on clinical characteristics without adequately addressing patient perspectives. Multiple underlying factors contribute to the persistence of PSPS-type 2, and effective pain management largely depends on identifying these factors, as the condition may be influenced by biological diversity, as well as psychological and social factors [8]. All the components result in patients experiencing lower quality-of-life scores, higher levels of pain and disability, increased psychological morbidity, and elevated rates of unemployment.

Therapeutic approaches to managing pain may include pharmacological, surgical, and non-surgical interventions [4,9,10]. As a non-surgical intervention, spinal cord stimulation (SCS) has demonstrated efficacy in managing persistent pain, with a lower morbidity rate compared to re-operation. Based on the pain gate control theory proposed by Ronald Melzack and Patrick D. Wall, SCS delivers pulsed electrical energy near the spinal cord by implanting electrodes in the relevant epidural space to modulate ascending pain signals [10,11,12,13]. In a case series with long-term follow-up, North et al. reported favorable outcomes for SCS therapy in patients with PSPS-type 2, showing success rates of 53–60% at 2.2 years and 47–54% at 5 years. In fact, improvements were noted in several clinical outcomes, including reduced pain severity (VAS scores), enhanced functional status (ODI scores), and decreased pain catastrophizing levels [4,10]. Although the gate control theory remains valid, current research indicates that the underlying mechanisms of SCS are complex and not fully understood. Evidence suggests that dorsal column stimulation may employ different mechanisms of analgesia when used for neuropathic versus ischemic pain. For example, the conditioned modulation of pain may be associated with the effect of SCS [14].

Furthermore, biological (nociceptive processing) and psychosocial factors such as emotional states, sociocultural context, behavioral and cognitive mechanisms, as well as demographic aspects (gender, depression, anxiety, and low self-esteem) contribute to the multidimensionality and heterogeneity of pain among patients [15,16,17,18]. Therefore, it is imperative to classify these factors to assess patient suitability for SCS treatment and optimize its effectiveness, as these elements influence patients’ physical functioning and health outcomes differentially [19]. While recent studies emphasize the relevance of psychosocial factors in patient care, these are infrequently documented, with most of the research focusing primarily on biological aspects. Psychological factors significantly influence patients’ pain perception, disability, and response to therapy [16,17,20,21]. Additionally, it is crucial to carefully examine psychosocial factors, as individuals with chronic pain frequently exhibit high levels of pain catastrophizing. This psychological phenomenon involves a persistent pattern of distressing cognitive and emotional responses to current or anticipated pain experiences. The proposed study aims to investigate whether psychosocial responses are associated with functionality and pain perception following the application of spinal cord stimulation treatment in patients with PSPS-type 2. Furthermore, this study aims to elucidate the relationship between catastrophizing, fear of movement, and central sensitization scores with regard to pain perception and disability.

## 2. Materials and Methods

### 2.1. Study Design

A cross-sectional descriptive and comparative study was conducted including a sample of patients diagnosed with PSPS-type 2. This study was approved by the Ethics Committee of Research with medicines in the Salamanca Health Area (Spain) (reference number: 2023 10 1435). All participants who chose to enroll in this study provided their agreement by signing an informed consent form prior to participation in accordance with the ethical guidelines of the Declaration of Helsinki [22]. The trial adhered to the STROBE (Strengthening the Reporting of Observational Studies in Epidemiology) guidelines in both its design and the progression of participants [23]. The STROBE guidelines comprise a 22-item checklist aimed at enhancing the quality and clarity of reporting in observational research. These guidelines address crucial elements of study methodology, design, and findings, enabling readers to critically evaluate a study’s credibility and validity. Primarily applicable to cross-sectional, cohort, and case–control studies, the STROBE checklist serves as a tool to ensure comprehensive and transparent reporting in epidemiological research.

### 2.2. Participants and Settings

This is a single-center study of patients diagnosed with spinal pain syndrome who are undergoing SCS at the Pain Unit at the Hospital of Salamanca (Spain). The participants were selected according to the compliance criteria established by the guidance of the Neurostimulation Appropriateness Consensus Committee (NACC) of neurostimulation practice [24]. All patients were informed of data procedures. The eligibility inclusion criteria were as follows: (i) a diagnosis of PSPS-type 2 with back pain and lower-limb pain caused by spine pathology; (ii) patients aged 18 years or older; (iii) ≥12 months with spinal cord stimulation; and (iv) a VAS score > 7. The exclusion criteria were as follows: (i) severe fractures or pathologies in the spine or lower limbs; (ii) oncological diseases; (iii) rheumatic diseases; and (iv) psychiatric disorders. All these cases were diagnosed following the initiation of spinal cord stimulation treatment.

### 2.3. Measures

The study participants were invited to attend a single session. In a face-to-face setting, the patients completed the information pertinent to this study. Participants responded to a series of questionnaires assessing anthropometric variables, clinical factors (disability and pain perception), psychosocial characteristics (TSK, PCS, CSI), as well as years of stimulation, which comprised both open-ended questions and validated instruments.

#### 2.3.1. Anthropometric Variables

Information about sex assigned at birth, age, and anthropometric measurements were assessed using a scale equipped with an integrated stadiometer. Additionally, the duration since the implantation of the spinal cord neurostimulator was recorded.

#### 2.3.2. Disability: Oswestry Disability Index (ODI)

The ODI stands as the most utilized and validated evaluation tool for assessing lumbar pain. This self-administered test comprises ten sections, each designed to evaluate limitations in everyday activities. The sections are scored from 0 to 5, with 5 indicating the highest level of instability, and there is a maximum possible score of 50 points [25]. To calculate the total rate, the participant’s score is divided by the maximum possible score and then multiplied by 100 to yield a percentage [26]. A higher percentage on the questionnaire signifies greater disability related to low back pain [27]. The ODI is widely considered the benchmark for assessing functional outcomes in low back conditions [28]. The Spanish version of the ODI has been validated on several occasions [29] and demonstrates high sensitivity and specificity in evaluating function [30,31].

#### 2.3.3. Perception of Pain: Visual Analog Scale (VAS)

The Visual Analog Scale (VAS) stands out as the most representative pain measurement tool and has emerged as the preferred choice due to its ease of comprehension and widespread adoption [32]. In the current study, a scale was employed ranging from 0 to 10. This instrument effectively quantifies pain in a subjective and selective manner, with 0 indicating no pain and 10 representing the most severe pain imaginable. The VAS surpasses descriptive and fixed-value scales in effectiveness. Research has demonstrated that the VAS possesses high reliability, with an alpha coefficient of 0.97 [33]. The patients were asked to indicate the pain they have when the question is performed, therefore at this moment of the day.

#### 2.3.4. Tampa Scale of Kinesiophobia (TSK)

Fear of movement or reinjury was evaluated using the TSK, a self-report questionnaire. This instrument consists of various questions rated on a 4-point Likert scale, ranging from “strongly disagree” to “strongly agree”. A higher score on the TSK indicates a greater fear of movement, while a lower score suggests a lesser fear of movement. The TSK demonstrated strong internal consistency, with scores up to α = 0.90, and an excellent test–retest reliability [34].

#### 2.3.5. Pain Catastrophizing Scale

This study will employ the Pain Catastrophizing Scale (PCS), a self-report questionnaire consisting of 13 items rated on a 0–4 Likert-type scale, to evaluate the degree of catastrophizing when experiencing pain [35]. The scale’s structure comprises three interconnected factors, helplessness, rumination, and magnification, which collectively represent a single second-order latent construct known as catastrophizing [36]. Scores on the PCS range from 0 to 52, with higher scores indicating greater levels of catastrophizing. Research has demonstrated that the PCS possesses satisfactory content and construct validity, internal consistency, and test–retest reliability across various musculoskeletal conditions [37,38] and in different language versions [39]. Specifically, the Spanish adaptation of the PCS exhibits an internal consistency of 0.79 and a test–retest reliability of 0.84 [40]. Lower scores on the scale suggest minimal catastrophizing, while higher scores indicate elevated levels of catastrophizing [40,41].

#### 2.3.6. Central Sensitization Inventory

The Central Sensitization Inventory (CSI) was used to distinguish and classify patients who might have symptoms related to a central sensitization syndrome [42] and to better assess the symptoms that may be associated with central sensitization [43]. The first part of the CSI consists of 25 items that are related to the patient’s current health symptoms. Each of these items is measured on a temporary 5-point Likert scale, with the following numerical rating scale: Never (0), Rarely (1), Sometimes (2), Often (3), and Always (4). This accumulates a score that can range from 0 to 100. The second part collects information on previously diagnosed central sensitization syndromes or their related conditions [43]. The CSI score is associated with all independent factors, with consistent findings on the relationship of the CSI with psychosocial and mental behaviors, as shown by multiple previous studies [44,45,46]. However, the score is not so much associated with the change in pain processing [47,48]. Thus, it appears that the CSI score is more positively correlated with psychological hypervigilance than the change in responsiveness of nociceptive neurons [47,49].

#### 2.3.7. Spinal Cord Stimulation

SCS was a therapeutic approach that employed an implantable pulse generator, offering the possibility of improved treatment outcomes through stimulation algorithms and settings [50]. For patients with chronic pain that is not effectively managed through conservative or less-invasive methods, SCS offers a reversible, neuromodulatory intervention [51]. By focusing on peripheral regions, such as the dorsal root ganglion, SCS provided more precise anatomical targeting for treatment [52]. Moreover, subthreshold stimulation, which utilized high-frequency or burst energy delivery, had the capacity to eliminate unwanted and off-target paresthesia. Research indicated that subthreshold stimulation at high frequencies and/or using alternative stimulation patterns yielded comparable or even better pain relief than conventional SCS. The technique involved inserting two octopolar electrodes through the epidural space and positioning them under the dorsal area, behind the posterior horn of the spinal cord. The chosen location was between the T8 and T11 vertebrae, where the highest synaptic activity of the spinothalamic tracts responsible for gathering painful sensations in the legs and lumbar region was concentrated [53].

### 2.4. Statistical Analysis

This study included all individuals who consented to data provision. For categorical variables, descriptive statistics were reported as frequencies and percentages. Continuous data following a normal distribution were summarized using means, 95% confidence intervals (CIs), interquartile ranges, and standard deviations (SDs). Relationships between study variables were examined through correlation analyses. Specifically, Pearson correlations were calculated, and multicollinearity was assessed by determining the variance inflation factor (VIF) and tolerance values for predictor variables. To test the study hypotheses, two linear regression analyses were conducted. The first linear regression utilized pain perception as the criterion variable, while the second regression employed disability as the criterion variable (incorporating age, weight, height, BMI, years of SCS, VAS, CSI, ODI, TSK, and PCS). Bias-corrected confidence intervals were computed using 5000 bootstrap resamples. Statistical significance was established at *p* < 0.05. Data analysis was performed using SPSS version 25 (IBM Corp., Armonk, NY, USA) and JASP application (Version 00.15.0.0.0) statistical software (2023). A researcher uninvolved in data collection and provided with coded data conducted the statistical analysis.

## 3. Results

### 3.1. Participation Flow and Sample Characteristics

**Figure 1:**
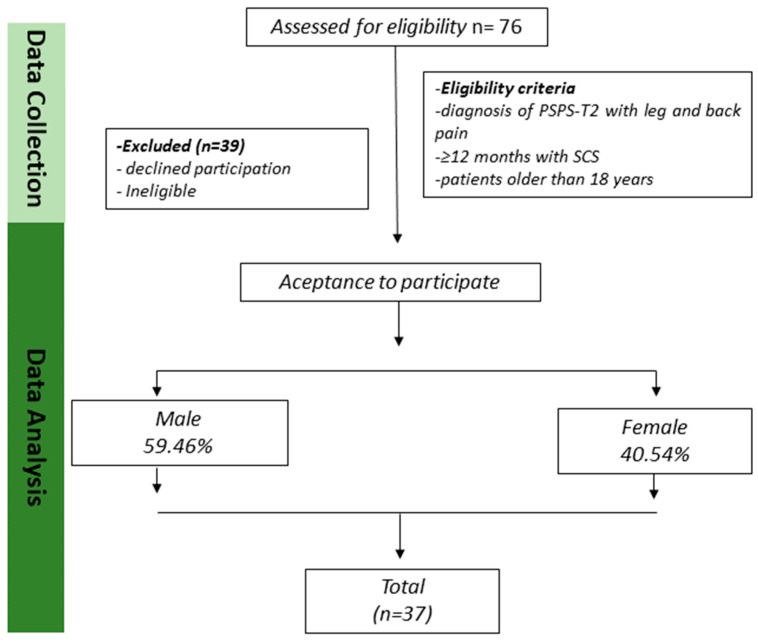
The design and progression of participants through the trial.

Between 28 March 2024 and 26 October 2024, an initial informational meeting was attended by 76 individuals with SCS. Subsequently, 19 were declined participation: 15 were ineligible and 5 did not provide a final decision. Ultimately, 37 patients were successfully enrolled in this study. Detailed information on the recruited patients is presented in Figure 1. The data pertaining to age, anthropometric characteristics, duration of spinal cord stimulation, and all clinical assessments implemented are presented in Table 1.

### 3.2. Main Outcomes

#### 3.2.1. Correlation Analysis

Table 2 displays the main findings, including Pearson correlation coefficients (r) and corresponding *p*-values for different variables. Statistical significance is determined by a *p*-value below 0.05, suggesting that the predictor is unlikely to be the result of chance and is therefore considered significant.

#### 3.2.2. Predicting Pain Perception and Disability

**Figure 2:**
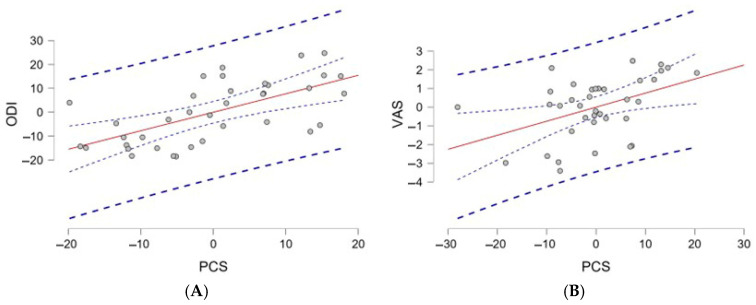
The image displays two scatter plots, labeled (**A**,**B**), that represent the relationships between the PCS (Pain Catastrophizing Scale) and two outcome variables: the ODI (Oswestry Disability Index) and VAS (Visual Analog Scale).

Table 3 and Table 4 display the outcomes of regression analyses predicting disability and pain perception. With regard to disability, the regression analysis indicated that the PCS was the only statistically significant predictor of the outcome variable, demonstrating a strong positive association (β = 0.90, *p* < 0.01). Conversely, both the CSI and age exhibited weak associations (β = 0.18, *p* = 0.14 CSI; β = 0.08, *p* = 0.32 age) and were not statistically significant predictors (Figure 2A). The model accounted for 94% of the variance (R^2^ = 0.94), suggesting that the PCS contributed substantially to the model’s explanatory power, whereas the CSI and age did not independently predict the outcome.

Regression analysis revealed that the PCS was the sole statistically significant predictor of pain perception (β = 0.075, t = 2.84, *p* = 0.008), accounting for a substantial portion of the variance explained by the model (R^2^ = 0.46, ΔR^2^ = 0.46, *p* = 0.01) (Figure 2B). The CSI and TSK were not significant predictors (*p* > 0.05), suggesting that the PCS was the primary contributor to the model, whereas the CSI and TSK did not independently influence the outcome.

## 4. Discussion

This research enhanced our comprehension of the mechanisms by which catastrophizing exhibited a positive and statistically significant link to disability. The model provided strong evidence for the PCS as a predictor of the outcome variable, emphasizing the significance of pain catastrophizing within this study’s framework. Nevertheless, the CSI and age lacked statistically significant effects. In terms of pain perception, the analysis revealed the PCS as the sole significant predictor among the variables examined, demonstrating that pain catastrophizing had a quantifiable impact on the outcome. Conversely, the CSI and TSK did not show statistically significant effects, suggesting these factors may not have independently affected the outcome variable in this context. This implies that targeting the reduction in catastrophizing thoughts could be crucial for enhancing outcomes. The outcomes of SCS are complex and vary depending on the type of pain. With regard to neuropathic pain, SCS appears to alter local neurochemistry by increasing the release of GABA and serotonin, which reduces hyperexcitability in neurons and suppresses excitatory cytokines like glutamate. In contrast, for ischemic pain SCS likely relieves pain by modulating sympathetic tone and restoring balance between oxygen supply and demand [54,55]. So, a therapeutic goal of employed SCS is considered useful if it includes a strict selection of a patients and a multidisciplinary approach [56]. Recent emphasis has been placed on patient-centered strategies as crucial elements in managing individuals with ongoing pain [57].

As a result, identifying high-risk factors can assist healthcare providers in recognizing patients who may be more prone to developing chronic pain. This knowledge enables the creation of comprehensive treatment strategies that address both the physical and psychological aspects of the condition. Firstly, there is no convincing evidence that the presence of a psychopathological disorder affects the predicted outcome of SCS therapy in patients with PSPS-type 2 [58]. However, high levels of kinesiophobia before surgery are linked to nonresponse in patients undergoing 10 kHz SCS therapy. Additionally, no responders show greater pain intensity, higher self-perceived disability, and more significant pain catastrophizing at baseline compared to responders [59]. In patients with chronic radicular pain following lumbar spine surgery, spinal cord burst stimulation did not show a significant difference compared to placebo stimulation regarding changes in self-reported back pain-related disability after spinal cord stimulator placement [60]. Therefore, it is important to consider the analgesic response generated by the placebo effect in treatment, as it can influence pain perception and the overall effectiveness of the therapy. Finally, to provide high-grade evidence for predictive factors, SCS studies of high quality are needed in which standardized factors predictive of SCS success, based on in-patient improvements, are monitored and reported [61].

This study’s clinical implications emphasize the necessity of addressing psychosocial constructs, particularly pain catastrophizing, when managing pain and disability in patients. The identification of the PCS as a key predictor for both disability and pain perception underscores the importance of integrating these elements into patient care. Interestingly, other factors such as the CSI, age, and the TSK were not found to significantly impact clinical outcomes to the same degree, despite their relevance. These findings have the potential to enhance clinical decision-making regarding SCS. To optimize the effectiveness of this neuromodulation therapy, it is crucial to carefully select appropriate patients [62]. Therefore, the recognition of pain catastrophizing as a significant predictive factor for disability and pain perception could assist clinicians in more accurately determining a patient’s suitability for this treatment. The results of this study highlight the need for further research to identify predictors of the successful outcomes in spinal cord stimulation [63]. In fact, assessing a patient’s pain catastrophizing level prior to SCS may help predict treatment success. This approach could not only increase the effectiveness of SCS but also optimize resource allocation and patient selection. In order to avoid interventions that may be less effective due to the lack of consideration of psychological factors from the outset, tailoring approaches to enhance the quality of life for patients experiencing persistent pain after spinal surgery may be particularly valuable when customized to individual clinical circumstances [64].

This study’s clinical implications underscore the necessity of addressing psychosocial constructs, particularly pain catastrophizing, in the management of pain and disability in patients. The identification of the PCS as a primary predictor for both outcomes emphasizes the importance of integrating these elements into patient care. Notably, other factors such as the CSI, age, and the TSK were not found to significantly impact clinical outcomes to the same extent, despite their relevance. These findings have the potential to enhance clinical decision-making regarding SCS. Based on these results, it is evident that to optimize the efficacy of this neuromodulation therapy, careful patient selection is crucial, as indicated by similar findings [62]. Therefore, the identification of pain catastrophizing as a significant predictive factor could potentially assist clinicians in more accurately determining a patient’s suitability for this treatment. The findings of this study underscore the necessity for further research to identify predictors of successful outcomes in spinal cord stimulation, as indicated in previous studies [63]. Indeed, evaluating a patient’s pain catastrophizing level prior to SCS may contribute to predicting treatment success. This approach could not only enhance the efficacy of SCS but also optimize resource allocation. Furthermore, tailoring approaches to improve the quality of life for patients experiencing persistent pain after spinal surgery may be particularly beneficial when customized to individual clinical circumstances [64].

### Limitations

It is crucial to recognize that the research design has certain drawbacks. One key limitation of cross-sectional studies is their inability to determine cause-and-effect relationships. This is because data are collected at only one point in time, making it impossible to establish causality between variables. The findings are dependent on the sample size and diversity used for analysis. To ensure generalizability, future research should replicate these findings with larger, more diverse samples. Given the correlation among predictors, future studies could explore interaction effects to better understand how these variables influence the outcome together, rather than in isolation. Validating the model with broader samples will be crucial for enhancing the reliability and applicability of these findings.

## 5. Conclusions

To summarize, this research underscores the critical role of pain catastrophizing in addressing pain and disability. This study’s identification of the PCS as a key predictor of clinical outcomes suggests that incorporating psychological evaluations and therapies could improve the efficacy of treatments such as SCS. While factors like central sensitization, age, and kinesiophobia were not found to have direct impacts, they may still be relevant in the broader scope of patient care.

## Figures and Tables

**Table 1:** Descriptive statistics (mean, standard deviation, and range) of the anthropometric variables analyzed.

Factors	Value (Mean ± SD)	Range
Age	54.73 ± 9.13	50–59
Gender, freq (%)	37	Male: 59.46% Female: 40.54%
Weight	79.67 ± 14.41	70–86
Height	1.69 ± 0.09	1.61–1.77
BMI	27.71 ± 4.38	24.69–30.12
Years SCS	4.68 ± 5.25	1–6
VAS	5.6 ± 1.96	5–7
CSI	42.08 ± 18.39	33–57
ODI	37.62 ± 16.13	24–52
TSK	33.11 ± 8.76	28–40
PCS	28.43 ± 13.14	18–39

Note: Gender, freq (%) (gender distribution of the participants); BMI (Body Mass Index); Years SCS (years of spinal cord stimulation); VAS (Visual Analog Scale); CSI (Central Sensitization Inventory); ODI (Oswestry Disability Index); TSK (Tampa Scale of Kinesiophobia); PCS (Pain Catastrophizing Scale).

**Table 2:** Correlation analysis.

Variables	Correlation Coefficient	
	Disability	Pain Perception
Age	−0.32 (0.05) *	−0.01 (0.93)
Weight	0.23 (0.15)	0.15 (0.34)
Height	0.30 (0.06)	−0.07 (0.66)
BMI	0.06 (0.69)	0.11 (0.48)
Years SCS	−0.43 (0.70)	−0.17 (0.32)
VAS	0.61 (<0.001) *	-----
CSI	0.52 (0.001) *	0.48 (0.002) *
ODI	-----	0.61 (0.001) *
TSK	0.30 (0.06)	0.41 (0.01) *
PCS	0.76 (0.01) *	0.65 (0.001) *

Note: Pearson correlation coefficients: r and *p*-value. Note. BMI (Body Mass Index); Years SCS (years of spinal cord stimulation); VAS (Visual Analog Scale); CSI (Central Sensitization Inventory); ODI (Oswestry Disability Index); TSK (Tampa Scale of Kinesiophobia); PCS (Pain Catastrophizing Scale). * *p* < 0.05, the predictor is considered statistically significant.

**Table 3:** Summary of the linear regression analysis predicting disability.

Step and Variable	R^2^	ΔR^2^	ΔF (*p*)	β	t (*p*)	95% CI for t
1. Control Variables	0.94	0.94	3–34 (<0.01)			
PCS				0.90	5.72 (<0.01) *	0.58–1.21
CSI				0.18	1.51 (0.14)	0.072–0.454
Age				0.08	0.99 (0.32)	−0.08–0.23

Note. CSI (Central Sensitization Inventory); PCS (Pain Catastrophizing Scale). R^2^ (R-squared); ΔR^2^ (Delta R-squared); ΔF (*p*): the F-test statistic; β (Beta); t (*p*): t-statistic and *p*-value and 95% CI for t (confidence interval). * *p* < 0.05, the predictor is considered statistically significant.

**Table 4:** Summary of the linear regression analysis predicting pain perception.

Step and Variable	R^2^	ΔR^2^	ΔF (*p*)	β	t (*p*)	95% CI for t
1. Control Variables	0.46	0.46	3–33 (0.01)			
PCS				0.075	2.84 (0.008) *	0.02–0.13
CSI				0.020	1.20 (0.22)	−0.01–0.05
TSK				0.044	1.300 (0.203)	−0.05–0.09

Note. CSI (Central Sensitization Inventory); TSK (Tampa Scale of Kinesiophobia); PCS (Pain Catastrophizing Scale). R^2^ (R-squared); ΔR^2^ (Delta R-squared); ΔF (*p*): the F-test statistic; β (Beta); t (*p*): t-statistic and *p*-value and 95% CI for t (confidence interval); * *p* < 0.05, the predictor is considered statistically significant.

## Data Availability

The data presented in this study are available on request from the corresponding author.
